# The nucleocapsid protein of Crimean–Congo hemorrhagic fever virus interacts with eIF4A to promote the translation of viral mRNA in cells

**DOI:** 10.1016/j.jbc.2025.110173

**Published:** 2025-05-04

**Authors:** Saima Ali, Songyang Ren, Alexis Agsaoa, Sheema Mir, Mohammad A. Mir

**Affiliations:** College of Veterinary Medicine, Western University of Health Sciences, Pomona, California, USA

**Keywords:** RNA virus, mRNA translation, Nucleocapsid protein, CCHF, viral mRNA translation

## Abstract

Crimean–Congo hemorrhagic fever virus (CCHFV) is a tick-borne nairovirus in the Bunyavirales order. Unlike many viral infections, CCHFV does not induce a host translation shutdown, posing the question of how its mRNAs are efficiently translated amidst competing host transcripts. Here, we show that the CCHFV nucleocapsid protein (N protein) enhances the translation of luciferase reporter mRNA with the help of the viral S-segment mRNA-derived 5′ UTR. Chemical inhibition of eIF4E did not affect the N protein–mediated preferential translation of the reporter mRNA. However, translation shutdowns caused by either proteolytic cleavage of eIF4G or chemical inhibition of eIF4A abolished the N protein–mediated preferential translation of the reporter mRNA. These findings demonstrate that the CCHFV N protein requires both eIF4A and eIF4G to facilitate mRNA translation with the assistance of the viral mRNA 5′ UTR. Randomization of the viral 5′ UTR significantly reduced the translation efficiency of viral S-segment mRNA in cells. Our results demonstrate that WT S-segment mRNA was heavily engaged with ribosomes, and N protein likely remained associated with the WT 5′ UTR, continuously facilitating ribosome loading, promoting polysome formation, and enhancing protein production. In contrast, most S-segment mRNA with a randomized 5′ UTR was largely free from ribosome engagement, explaining the lower protein production from this transcript. Our results demonstrate that the N protein binds to eIF4A and likely reserves a population of eIF4A–eIF4G complexes that remain dedicated to selectively boost the translation of viral S-segment mRNA, thus avoiding competition from host cell transcripts for the same translation machinery.

Crimean-Congo hemorrhagic fever (CCHF) is a severe viral disease caused by a tick-borne nairovirus in the Bunyavirales order (1). It is endemic in many regions, including Africa, the Balkans, the Middle East, and parts of Asia, with a mortality rate of up to 40% (2, 3, 4, 5). Although CCHF is not currently endemic in the United States, it poses a potential threat due to global travel and the presence of tick vectors that could potentially carry the virus (6).

In the U.S., the primary concern regarding CCHF is for military personnel, healthcare workers, and those traveling to endemic areas (7). The virus spreads through tick bites, contact with infected animal blood or tissues, and human-to-human transmission *via* bodily fluids (8, 9). There is no vaccine available, and treatment mainly involves supportive care, although the antiviral drug Ribavirin has shown some effectiveness in managing the disease. The development of mRNA vaccine against CCHF has been proposed (10) and multiple therapeutic targets have been suggested (11).

Recent frequent outbreaks in Mediterranean countries are likely due to the extensive habitat and population size of the CCHFV tick vector, which may be influenced by climate change (12). The CCHFV genome consists of three negative-sense RNA segments (S, M, and L) that encode the nucleocapsid protein (N protein), glycoprotein precursor, and RNA-dependent RNA polymerase, respectively (13). Although N protein is primarily seen as a crucial part of the viral capsid, recent research on the Lassa fever virus (Arenaviridae) and Sin Nombre hantavirus (SNV) (Hantaviridae) has uncovered N protein's diverse roles in the viral replication cycle (14, 15, 16, 17, 18). X-ray crystallography at 2.3-Å resolution recently revealed a unique metal-based endonuclease activity in CCHFV-N protein (19, 20, 21). Additionally, the interaction between N protein and cellular heat shock protein HSP70 has been implicated in CCHFV replication (22). Recently, HuR, an RNA-binding protein that enhances mRNA stability, has been reported to regulate the CCHFV minigenome and hazara virus replication by associating with viral genome (23). In addition, the nucleocapsid protein-specific affimer has been reported to inhibit virus replication (24). Cryo-EM reconstruction studies have recently shown a ring-shaped structure of the CCHFV-N protein-RNA oligomer, indicating that the interaction between the head and stalk domains of CCHFV-N protein leads to N protein multimerization while encapsidating the viral genome.

The rapid synthesis of viral proteins and replication of the viral genome within host cells are essential for the swift dissemination of new virions to neighboring cells during infection. Therefore, the efficiency of viral protein synthesis can influence the viral load in infected hosts and, consequently, the severity of the disease. Viruses have developed various strategies to enhance the translation of their mRNAs, thereby reducing competition with host cell transcripts for the translation machinery (25). For example, we previously reported that nucleocapsid protein of hantaviruses, another member of Bunyavirales order, specifically binds to the mRNA 5′ cap and ribosomal protein S19, a component of the 40S ribosomal subunit (17, 26). This binding allows the N protein to engage the 40S ribosomal subunit at the mRNA 5′ cap independent of the eIF4F cap-binding complex (26). The eIF4F complex, composed of eIF4A, eIF4G, and eIF4E, recruits the 43S preinitiation ribosome complex to the mRNA 5′ cap during canonical host translation process (27). We demonstrated that the hantavirus translation strategy preferentially enhances the translation of mRNA containing the viral mRNA 5′ UTR (28).

We also demonstrated that CCHFV N protein specifically binds to the viral mRNA 5′ UTR with its stalk domain (29). Recently, the amino acid residues of nairovirus nucleocapsid protein facilitating the RNA binding have been identified (30). Similar to hantavirus N protein, the CCHFV N protein aids the translation with the assistance of viral mRNA 5′ UTR (31). However, unlike hantavirus, the formation of the eIF4F complex is not necessary. Instead, the structural integrity of individual components of the eIF4F complex, particularly eIF4G, is essential for the CCHFV N protein–mediated translation mechanism. Here in this manuscript, we show that CCHFV N protein requires both eIF4A and eIF4G but not eIF4E to facilitate the mRNA translation. CCHFV N protein binds to eIF4A through its head domain. Our results suggest that CCHFV N protein likely reserves a population of eIF4A–eIF4G complexes that remain dedicated for the translation of viral mRNA by efficiently engaging host ribosomes on viral transcripts with the assistance of viral mRNA 5′ UTR. The efficient ribosome engagement significantly promoted the formation of polysomes on viral mRNAs.

## Results

### CCHFV N protein does not require eIF4E to facilitate the mRNA translation with the assistance of viral mRNA 5′UTR

**Figure 1:**
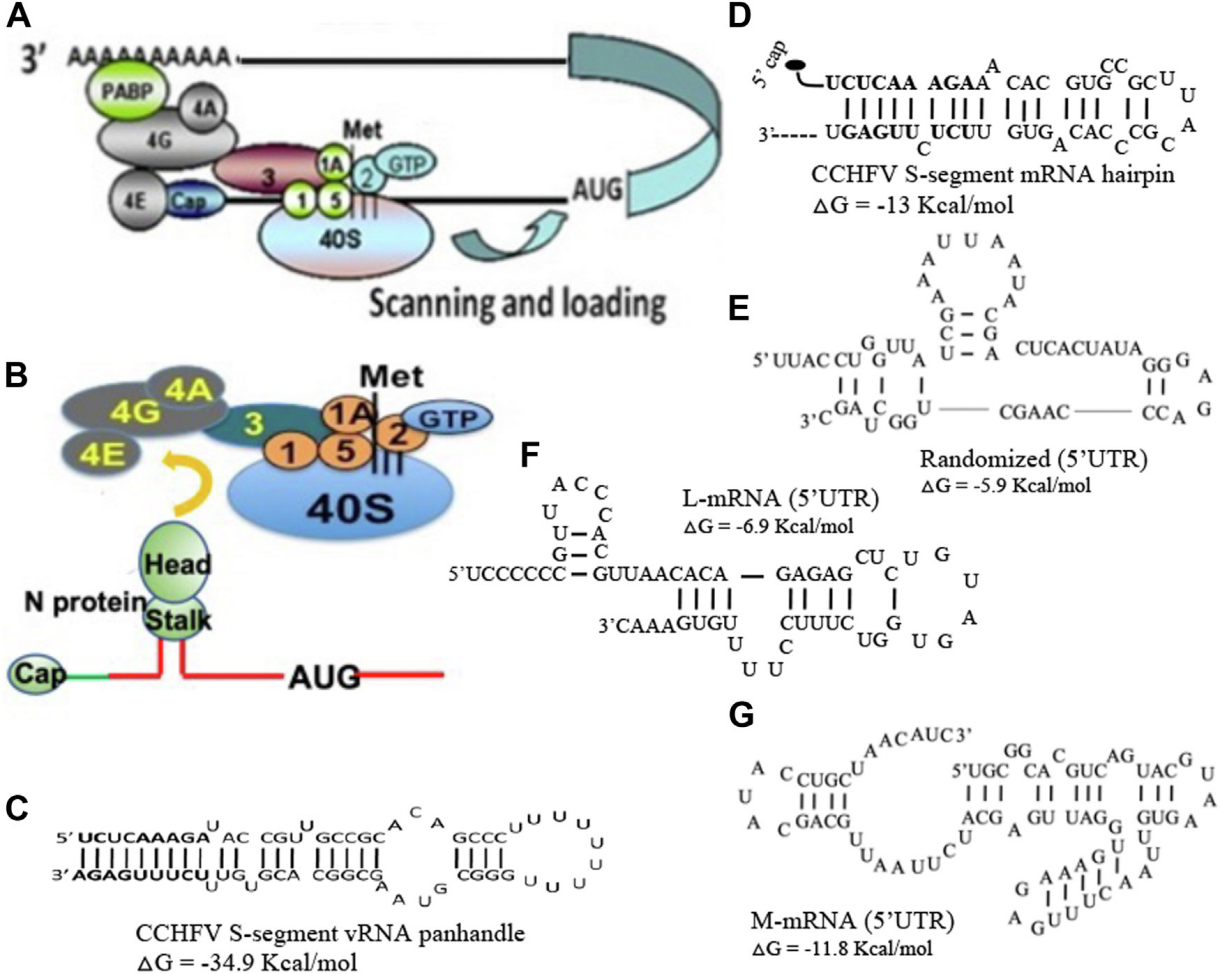
**Models of eukaryotic translation initiation and secondary structures of vRNA panhandles.***A*, eukaryotic translation initiation, showing the formation of eIF4F complex at the mRNA 5′ cap. Some initiation factors are not shown for simplicity. The initiation factors eIF4E, 4G, and 4A assemble and form eIF4F complex on the mRNA 5′ cap, at which 40S ribosomal subunit is recruited by interaction between eIF4G and eIF3. Poly A binding protein (PABP) helps to circularize the mRNA, as shown. *B*, a hypothetical model showing the binding of CCHFV N protein to S-segment mRNA hairpin structure through its stalk domain. Formation of eIF4F complex is not required but structural integrity of its structural components, especially eIF4G is required for this mechanism. *C*, panhandle structure formed by the partially complementary nucleotides at the 5′ and 3′ termini of CCHFV S-segment vRNA noncoding region, separated by a Uracil loop. *D*, hairpin structure formed by the 5′ UTR of CCHFV S-segment mRNA. The secondary structures formed by the randomized S-segment mRNA 5′UTR (*E*), the L-segment mRNA 5′ UTR (*F*), and M-segment mRNA 5′ UTR (*G*) are shown. The secondary structures were obtained by folding the RNA sequences using *mfold*. Folding parameters included the temperature of 37 °C and salt concentration of 1M NaCl.

The majority of eukaryotic mRNA translation is m7G cap-dependent and is initiated by the assembly of eIF4F cap-binding complex, composed of three initiation factors eIF4E, eIF4A (32, 33), and eIF4G (34), at the mRNA 5′ cap (Fig. 1*A*). Despite the tight regulation of eukaryotic translation, viruses have evolved selfish strategies to favor the translation of viral mRNA in the host cell during the course of infection. We recently reported that CCHFV N protein has two distinct RNA-binding sites in the stalk and head domains (29, 35). The RNA-binding site in the stalk domain specifically binds to the panhandle structure formed by the base pairing of complementary nucleotides at the 5′ and 3′ termini of the viral genome (Fig. 1*C*). The RNA-binding site in the head domain nonspecifically binds to the single strand RNA. Interestingly, unlike the 5′ UTR of L- and M-segment mRNA (Fig. 1, *E* and *F*), the 5′UTR of CCHFV S-segment mRNA folds into a hairpin structure (Fig. 1*D*) that resembles the panhandle structure formed by the vRNA. The S-segment mRNA 5′ UTR is also specifically recognized by the stalk domain of N protein (31). The specific interaction between N protein and viral mRNA 5′ UTR selectively favors the translation of downstream ORF in 5′ cap-independent manner (31). We previously reported that CCHFV N protein–mediated translation strategy does not require the assembly of eIF4F cap-binding complex but the individual components of this complex, especially eIF4G, are required for this viral translation mechanism (Fig. 1*B*) (31). It is critical to determine how CCHFV N protein with the assistance of viral mRNA 5′ UTR utilizes the components of eIF4F complex to facilitate the translation of downstream ORF. We focused to examine the potential role of eIF4E, eIF4G, and eIF4A in CCHFV N protein–mediated translation strategy.

**Figure 2:**
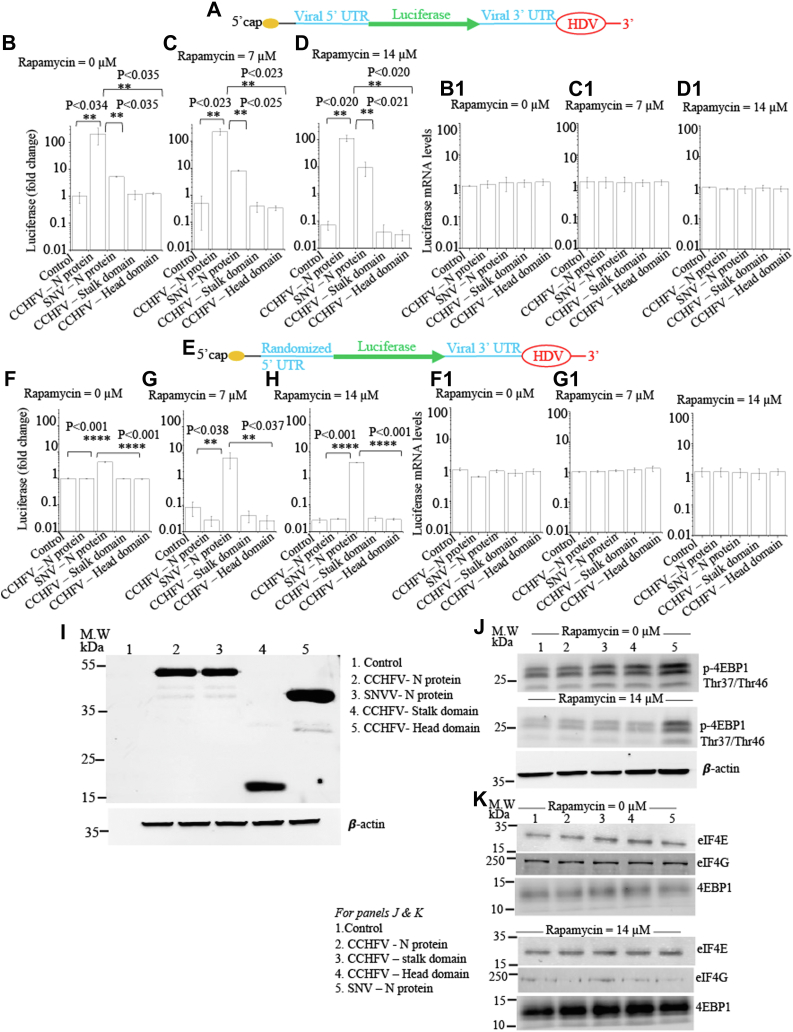
**CCHFV N protein does not require eIF4E to initiate mRNA translation.***A*, pictorial representation of the luciferase mRNA structurally mimicking the CCHFV S-segment mRNA, expressed from pCDLuc3 plasmid in transfected cells. *B*–*D*, fold change in luciferase activity in mock transfected cells (control) or cells transfected with plasmids expressing CCHFV N protein, SNV N protein, stalk domain of CCHFV N protein, or head domain of CCCHFV N protein, as shown. Luciferase activity was monitored in untreated cells (*B*) or cells treated with either 7 μM (*C*) or 14 μM (*D*) Rapamycin. The luciferase signal in (*B*–*D*) was normalized to the control in (*B*). It must be noted that cells in the control express the luciferase reporter but do not express any viral protein. *B1*, *C1*, and *D1*, total RNA in (*B*–*D*) was purified and luciferase mRNA was quantified by real time PCR analysis and shown in corresponding (*B1*–*D1*), respectively. Again, the mRNA levels in (*B1*–*D1*) were normalized to the control in (*B1*). *E*, pictorial representation of the luciferase mRNA expressed from pCDLuc4 plasmid in transfected cells. This mRNA is similar to the mRNA shown in (*A*) except the 5′ UTR sequence was randomized. *F*–*H*, the experiment in (*F*–*H*) was done exactly as (*B*–*D*), respectively, except the mRNA shown in (*A*) was replaced with the mRNA shown in (*E*). *F1*–*H1*, total RNA in (*F*–*H*) was purified and luciferase mRNA was quantified by real time PCR analysis and shown in corresponding (*F1*–*H1*), respectively. Normalization of the mRNA levels was done as mentioned above. *I*, the experiment in (*D*) was repeated in 6-well plates and cells were lysed 12 h after the treatment with Rapamycin (14 μM) and examined by Western blot analysis, using anti-His tag antibody. Shown are the C-terminally His-tagged fusion proteins expressed from transfected plasmids. *J*, the experiment in (*B* and *D*) was repeated in 6-well plates. Cells were lysed and examined by Western blot analysis, using anti-p-4EBP1 antibody (Cell Signaling Technologies, Cat# 2855). Shown are the levels of phosphorylated 4EBP1 in cells. *K*, the experiment in (*J*) was repeated. The cell lysates were pulldown using m7G Sepharose. The pulldown material was examined by Western blot analysis using antibodies against eIF4E, eIF4G, and 4EBP1, as mentioned in Experimental prcodeures. ∗∗>95% statistically significant, ∗∗∗∗>99% statistically significant. Note: The *p*-values were calculated by student's *t* test.

HEK293T cells in 96-well plates were transfected with plasmids expressing either WT CCHFV N protein or its stalk domain or head domain or SNV N protein, as control. SNV N protein facilitates the translation of capped mRNAs without the requirement of eIF4E, eIF4G, and eIF4A, as previously reported (17). Thirty-six hours post transfection, cells were again transfected with either PCDLuc3 or PCDLuc4 reporter plasmids (see Experimental procedures for details). The PCDLuc3 expresses luciferase mRNA structurally mimicking the CCHFV S-segment–derived mRNA (Fig. 2*A*). It contains the affimer viral S-segment mRNA-derived 5′ UTR sequence and 3′ UTR sequence truncated by the precisely incorporated hepatitis delta virus ribozyme (HDV) sequence. The 14-nucleotide sequence upstream of the 5′ UTR represents the cap-snatched sequence during virus infection to host cells. The PCDLuc4 reporter plasmid expresses the similar mRNA except the 5′ UTR sequence was randomized (Fig. 2*E*). Five hours post transfection with these luciferase reporter plasmids, the cells were incubated overnight with increasing concentrations of rapamycin, followed by the examination of luciferase signal and expression of viral proteins from transfected plasmids, as mentioned in Experimental procedures. It is evident from Figure 2*I* that all the proteins from transfected plasmids are well expressed in cells. It is well known that rapamycin interferes with mTOR signaling and inhibits the phosphorylation of 4E binding protein-1 (4EBP1). The unphosphorylated 4EBP1 binds and sequesters eIF4E, impairing the canonical host translation machinery. As shown in Figure 2*J*, the rapamycin treatment inhibited the phosphorylation of 4EBP1. The m7G Sepharose pulldown experiment (see Experimental procedures) revealed that unphosphorylated 4EBP1 in rapamycin-treated cells sequestered the eIF4E and diminished the interaction between eIF4E and eIF4G (Fig. 2*K*). It is evident from Figure 2, *B*–*D* that rapamycin inhibited the translation of luciferase reporter mRNA, structurally mimicking the viral S-segment mRNA, in dose-dependent manner in both mock-transfected control cells and cells expressing the head and stock domains of CCHFV N protein. The co-expression of WT CCHFV N protein and SNV N protein not only promoted the translation of reporter mRNA but also resisted the rapamycin-induced translation shut down (compare Fig. 2*B* with Fig. 2, *C* and *D*). We did not observe any noticeable change in the intrinsic steady-state levels of luciferase reporter mRNA, confirmed by real time PCR analysis (Fig. 2, *B1*–*D1*). When the experiment was similarly repeated with luciferase reporter mRNA having randomized viral mRNA 5′ UTR (Fig. 2*E*), the WT CCHFV NP unlike SNV N protein not only failed to promote the translation of reporter mRNA but also failed to inhibit the rapamycin-induced translation shut down (Fig. 2, *F*–*H*). Again, we did not observe any change in the intrinsic stead-state levels of reporter mRNA (Fig. 2, *F1*–*H1*). Taken together, the results from Figure 2 demonstrate that CCHFV N protein does not require eIF4E to promote the translation of reporter mRNA with the assistance of viral mRNA 5′UTR. In comparison, the SNV N protein facilitated the translation of luciferase reporter mRNAs in eIF4E-independent manner, irrespective of viral mRNA 5′ UTR, consistent with previously reported similar observations (17).

### CCHFV N protein–mediated translation initiation strategy requires eIF4A

**Figure 3:**
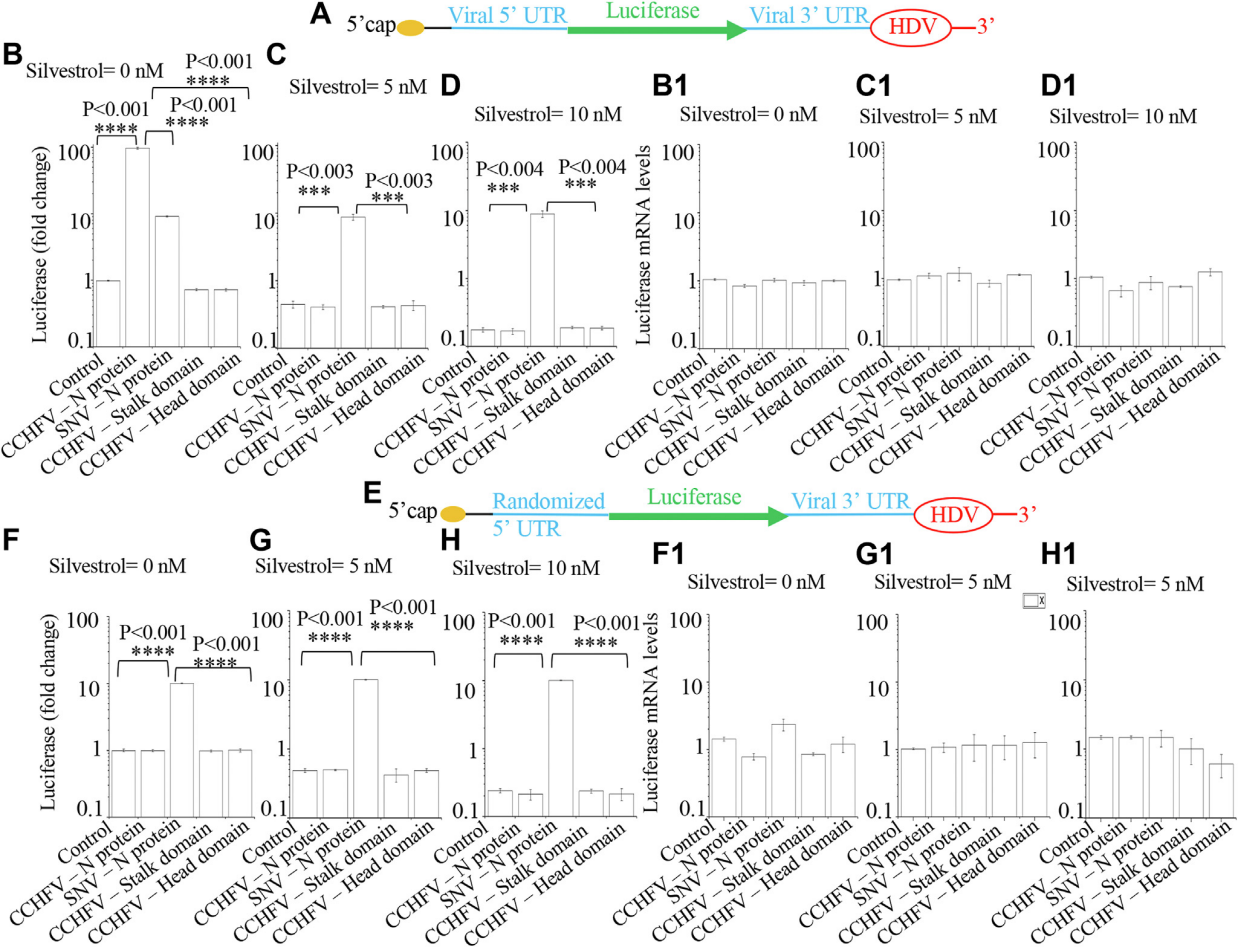
**Requirement of eIF4A for CCHFV N protein-mediated translation strategy.***A*, pictorial representation of the same luciferase mRNA as shown in Figure 2*A*. *B*–*D*, the experiment in (*B*–*D*) was done exactly as shown in Figure 2 (*B*–*D*), except the Rapamycin was this time replaced with silvestrol. The concentrations of silvestrol used are shown in the figure. *B1*, *C1*, and *D1*, real time PCR analysis showing the relative luciferase mRNA levels in the samples from (*B*–*D*), respectively. The mRNA levels in each sample were normalized related to the control in (*B1*). *E*, pictorial representation of the same mRNA as shown in Figure 2*E*. *F*–*H*, the experiment in (*F*–*H*) was carried out similar to (*B*–*D*), except the mRNA shown in (*A*) was replaced with the mRNA shown in (*E*). *F1*, *G1*, and *H1*, real time PCR analysis showing the relative luciferase mRNA levels in the samples from (*F*–*H*), respectively. The mRNA levels in each sample were normalized related to the control in (*F1*). ∗∗∗>99.5% statistically significant, ∗∗∗∗>99.9% statistically significant. Note: The *p*-values were calculated by student's *t* test.

To investigate the potential role of eIF4A in the CCHFV N protein–mediated translation mechanism, the experiment was repeated exactly as in Figure 2, but this time, the cells were treated with silvestrol instead of rapamycin. Silvestrol is a natural compound isolated from the plant *Aglaia foveolata* and belongs to the flavaglines, characterized by a cyclopenta[b]benzofuran skeleton (36). Identified as a specific inhibitor of the DEAD-box helicase eIF4A, silvestrol hinders the eIF4A helicase (37), which typically unwinds secondary structures at the mRNA 5′-UTR to facilitate the binding of the ribosomal pre-initiation complex (43S PIC). By stalling eIF4A at its mRNA substrate, silvestrol depletes eIF4A from the eIF4F cap-binding complex, thus inhibiting translation (38). It is evident from Figure 3 that except the stalk and head domains of CCHFV N protein, the co-expression of both WT CCHFV N protein and SNVV N protein facilitated the translation of luciferase mRNA, structurally mimicking the viral S-segment mRNA (Fig. 3*B*). However, the treatment with silvestrol inhibited the translation of luciferase mRNA in dose-dependent manner, except in cells co-expressing SNV N protein (Fig. 3, *C* and *D*). No changes in the luciferase mRNA levels were observed (Fig. 2, *B1*–*D1*). This demonstrates that unlike SNV N protein, the CCHFV N protein requires the eIF4A to facilitate the mRNA translation.

Next, the experiment was repeated with luciferase mRNA harboring the randomized 5′ UTR (Fig. 3*E*). Unlike SNV N protein, the CCHFV N protein and its head or stalk domains failed to facilitate the translation of luciferase mRNA harboring the randomized 5′ UTR (Fig. 3*F*). This further confirms that WT CCHFV N protein requires the viral mRNA 5′ UTR to facilitate the translation of downstream ORF. Again, the treatment with silvestrol inhibited the translation of luciferase mRNA in dose-dependent manner, except in cells expressing SNV N protein (Fig. 3, *G* and *H*). Again, no changes in mRNA levels were observed (Fig. 3, *F1*–*H1*). Taken together, these results clearly demonstrate that unlike SNV N protein, the CCHFV N protein requires the eIF4A to facilitate the mRNA translation with the assistance of viral mRNA 5′ UTR.

### Requirement of eIF4G for CCHFV N protein–mediated translation strategy

**Figure 4:**
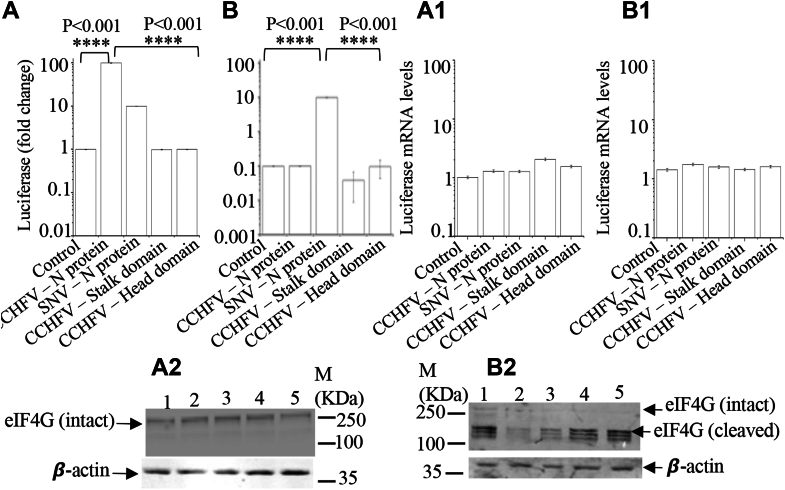
**Requirement of eIF4G for CCHFV N protein-mediated translation strategy.***A* and *B*, HEK293T cells were either mock transfected (control) of transfected with plasmids expressing CCHFV N protein, SNV N protein, stalk domain of CCHFV N protein, or head domain of CCCHFV N protein, as shown. Thirty-six hours post-transfection, cells were again transfected with either pCDLuc3 plasmid (*A*) or cotransfected with plasmids pCDLuc3 and pF/HRV-16 2A (*B*). Luciferase signal was monitored 24 h post transfection; the signal was normalized related to the control in (*A*) and plotted. *A1* and *B1*, real time PCR analysis showing the relative luciferase mRNA levels in (*A* and *B*), respectively. The mRNA levels were normalized related to the control in (*A*). *A2* and *B2*, Western blots showing the degradation of eIF4G in cells expressing 2A-protease. The experiments shown in (*A* and *B*) were repeated, and the resulting cell lysates were analyzed *via* Western blot using an anti-eIF4G antibody, as presented in (*A2* and *B2*), respectively. The lanes 1 to 5 represented mock transfected (control), transfected with plasmids expressing CCHFV N protein, SNV N protein, stalk domain of CCHFV N protein, or head domain of CCCHFV N protein, respectively, as shown in (*A* and *B*). ∗∗∗∗>99% statistically significant.

To delineate the role of eIF4G in the CCHFV N protein–mediated translation mechanism, we utilized human rhinovirus-16 2A protease to specifically cleave eIF4G in cells and assess whether this cleavage impacts the CCHFV N protein–mediated translation strategy. The specific cleavage of eIF4G by 2A protease, which leads to the inhibition of cap-dependent canonical host translation machinery, is well understood (39). Briefly, HEK293T cells were either mock transfected or transfected with plasmids expressing CCHFV N protein, SNV N protein, or the stalk or head domains of CCHFV N protein. Thirty-six hours post-transfection, cells were again cotransfected with PCDLuc3 plasmid, expressing luciferase mRNA structurally mimicking S-segment–derived mRNA, along with another plasmid expressing human rhinovirus-16 2A protease. Luciferase activity was examined 24 h post-transfection. As shown in Figure 4*A*, both CCHFV N protein and SNV N protein facilitated the translation of reported mRNA in cells deficient in 2A protease expression. However, co-expression of 2A protease inhibited the translation of reporter mRNA in cells expressing either WT CCHFV N protein or its stalk or head domains but failed to do so in cells expressing SNV N protein. This clearly demonstrates that, unlike SNV N protein, the CCHFV N protein requires eIF4G to facilitate mRNA translation, consistent with similar previously reported observations (31). An examination of cell lysates by Western blot analysis confirms the cleavage of eIF4G by the expression of 2A protease (Fig. 4, *A2* and *B2*).

### CCHFV N protein binds to eIF4A through its head domain

**Figure 5:**
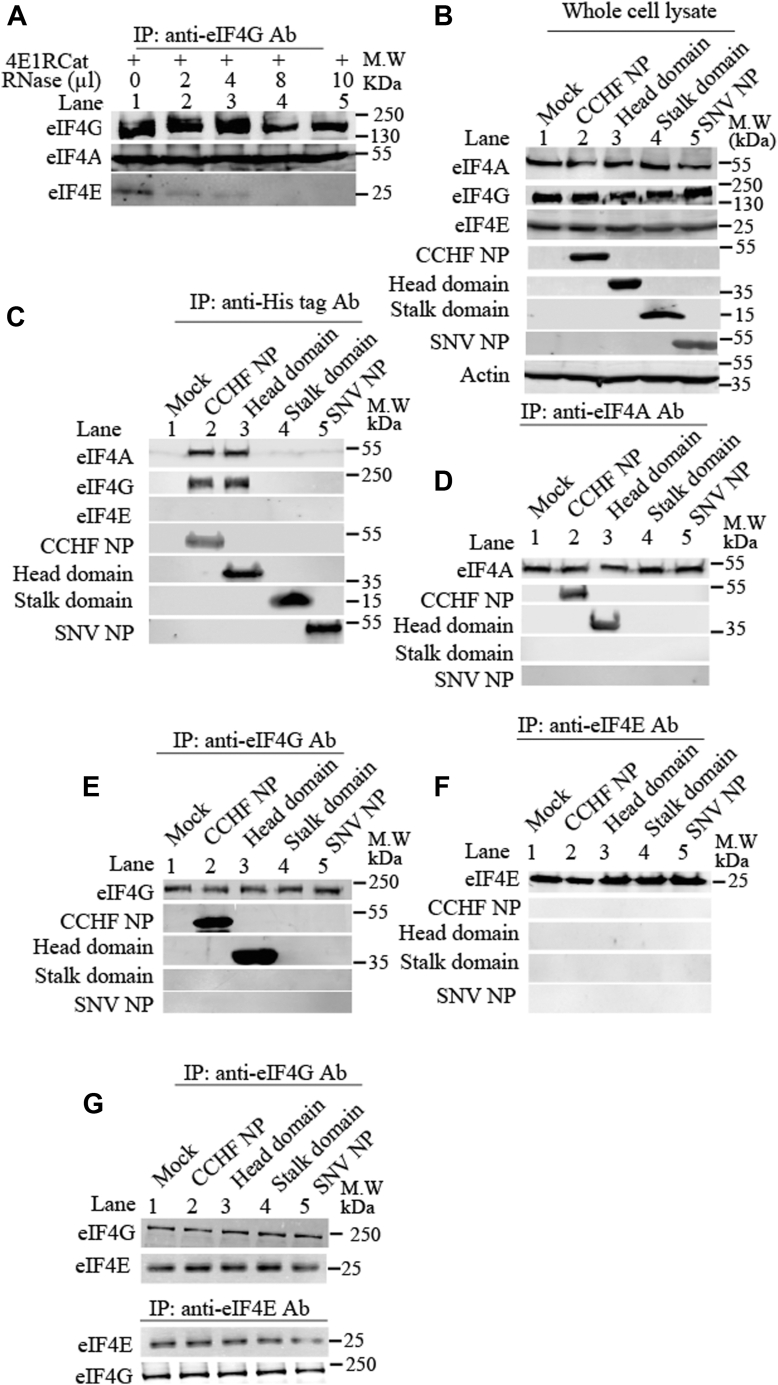
**Binding of CCHFV N protein to eIF4A.***A*, HEK293T cells grown in 10 cm dishes were lysed with 1.5 ml of RIPA buffer. Three hundred microliters of the resulting lysate was treated with a fixed concentration of 4E1RCat (30 μM) and increasing volumes of RNase A solution (2 μg/μl), as shown. The lysates were immunoprecipitated with anti-eIF4G antibody (Cell signaling technologies, Cat# 2469S) and the immunoprecipitated material was examined by Western blot using the appropriate antibody as shown. *B*, HEK293T cells were either mock transfected or transfected with plasmids expressing the C-terminally His-tagged recombinant proteins as shown in lanes 2 to 5. The cell lysates were examined by Western blot using the appropriate antibodies as shown. CCHF N protein, head, stalk domains, and SNV N protein were examined using anti-His tag monoclonal antibody. *C*–*F*, HEK293T cells were transfected with plasmids, followed by cell lysis as mentioned in (*B*). The lysates were treated with 4E1RCat (30 μM) and 8 μl of RNase A (2 μg/μl), followed by immunoprecipitation with either anti-His tag antibody (*C*) or anti-eIF4A antibody (*D*) or anti-eIF4G (*E*) or anti-eIF4E (*F*). The immunoprecipitated material was examined by Western blot analysis using appropriate antibodies, as shown. Note: CCHFV NP and SNV NP represent CCHFV N protein and SNV N protein, respectively. *G*, cell lysates without the treatment with 4E1RCat were immunoprecipitated with either anti-eIF4G or anti-eIF4E antibody, followed by the Western blot analysis of the immunoprecipitated material using appropriate antibodies.

Since CCHFV N protein requires both eIF4A and eIF4G to facilitate the mRNA translation with the assistance of viral mRNA 5′ UTR, we next wanted to determine whether N protein directly interacts with any components of the eIF4F complex. The eIF4G serves as bridge between eIF4A and eIF4E during the formation of eIF4F complex at the mRNA 5′ cap (Fig. 1*A*); it is necessary to dissociate the eIF4F complex in order to identify the exact component that selectively binds to CCHFV N protein. The HEK cell lysates were treated with chemical inhibitor 4E1RCat that inhibits the interaction between eIF4E and eIF4G (40, 41, 42), followed by increasing concentrations of RNase A to degrade the cellular RNA. The resulting solution was immunoprecipitated with anti-eIF4G antibody and the immunoprecipitated material was examined by Western blot analysis using anti-eIF4A and anti-eIF4E antibodies, as mentioned in Experimental procedures. It is evident from Figure 5*A* that treatment with 4E1RCat and RNAse A prevented the interaction with eIF4E but failed to disrupt the interaction between eIF4A and eIF4G in the lysates.

To identify the exact component of eIF4F complex that might bind to CCHFV N protein, HEK293T cells were either mock transfected or transfected with plasmids expressing C-terminally His-tagged CCHFV N protein, SNV N protein, or the stalk or head domains of CCHFV N protein. Cells were lysed 36 h post-transfection, and half of the cell lysates were examination by Western blot analysis which revealed the significant expression of proteins from transfected plasmids (Fig. 5*B*). The remaining of the lysates were treated with 4E1RCat and RNase A for 1 h at room temperature, followed by immunoprecipitated using either anti-His tag antibody (Fig. 5*C*) or anti-eIF4A antibody (Fig. 5*D*) or anti-eIF4G antibody (Fig. 5*E*) or anti-eIF4E antibody (Fig. 5*F*). The immunoprecipitated material was examined by appropriate antibodies. It is evident from Figure 5, *C*–*F* that both CCHFV N protein and its head domain copurified with both eIF4A and eIF4G. To determine whether CCHFV N protein competed with eIF4E for binding to eIF4G, cell lysates without the treatment with 4E1RCat were immunoprecipitated with either anti-eIF4G or anti-eIF4E antibody and the immunoprecipitated material was examined by Western blot analysis. It is evident from Figure 5*G* that none of the proteins expressed from transfected plasmids competed with eIF4E for binding to eIF4G (compare lanes 1 with lanes 2–5 in Fig. 5*G*).

**Figure 6:**
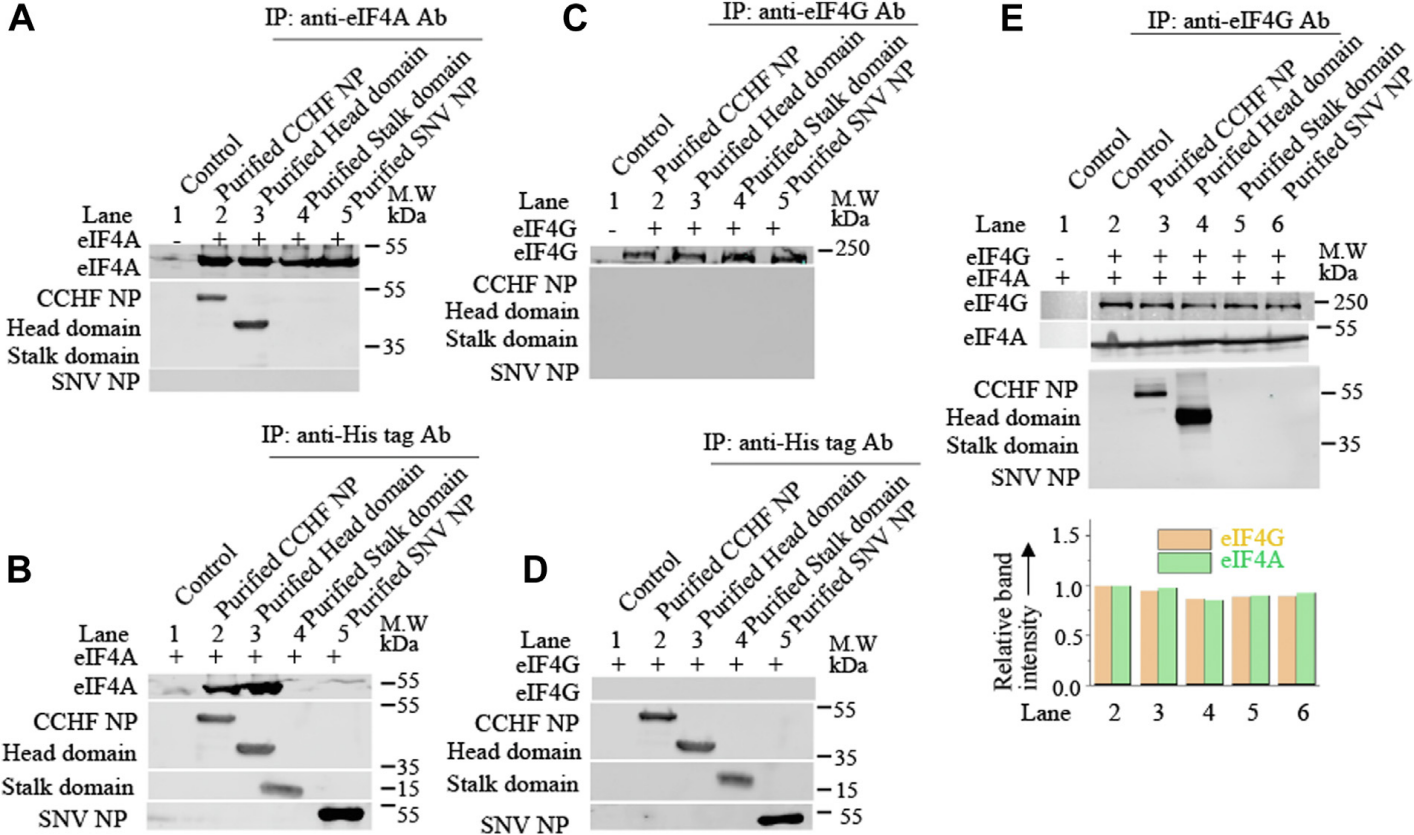
**Use of purified translation initiation factors to verify the N protein–eIF4A interaction.***A*, the purified eIF4A was incubated with purified CCHFV N protein (lane 2), purified head domain (lane 3), purified stalk domain (lane 4), and purified SNV N protein (lane 5) in 1× PBS. Lane 1 did not contain eIF4A but contained a mixture of CCHFV N protein, head domain, stalk domain, and SNV N protein. The protein mixtures were immunoprecipitated with anti-eIF4A antibody, followed by Western blot analysis of the immunoprecipitated material using either anti-His tag antibody or anti-eIF4A antibody, as shown. *B*, the purified proteins were mixed as mentioned in (*A*), except lane 1 had only eIF4A without any N protein. Immunoprecipitation was carried out using anti-His tag antibody, followed by Western blot analysis using either anti-eIF4A antibody or anti-His tag antibody, as shown. *C* and *D*, the experiment in (*C* and *D*) was carried out similar to (*A* and *B*), except eIF4A and anti-eIF4A antibody was replaced with eIF4G and anti-eIF4G antibody, as shown. *E*, the experiment in (*E*) was carried out similar to (*C*), except both eIF4A and eIF4G were used in the reaction, as shown. It must be noted that lane 1 contained only eiF4A and eIF4G without any viral protein. The band intensities of eIF4G and eIF4A were quantified, normalized to first band on the *left*, and plotted in the bar graph.

Since eIF4A and eIF4G form a stable complex, it was necessary to determine whether the head domain of CCHFV N protein binds to eIF4A or eIF4G or both. The SNV N protein, CCHFV N protein, its head and stalk domains were expressed and purified in bacteria, as mentioned in Experimental procedures. Bacterially expressed and purified eIF4A and eIF4G were obtained from ORIGENE, as mentioned in Experimental procedures. The purified eIF4A was incubated with either purified SNV N protein or CCHFV N protein or head domain or stalk domain in 1× PBS at a final concentration of 143 μM each, for 1 h at room temperature. The resulting mixture was immunoprecipitated with anti-eIF4A antibody (Fig. 6*A*), followed by the examination of immunoprecipitated material by Western blot analysis using anti-His tag antibody. It is evident from Figure 6*A* that both WT CCHFV N protein and its head domain copurified with eIF4A. The interaction between eIF4A and CCHFV N protein through its head domain was confirmed by reverse-immunoprecipitation method (Fig. 6*B*). This experiment was exactly repeated with eIF4G by replacing the eIF4A with eIF4G in the immunoprecipitation analysis (Fig. 6, *C* and *D*), which demonstrated that eIF4G did not show interaction with any tested protein. However when both eIF4G and eIF4A were incubated together with either purified SNV N protein or CCHFV N protein or head domain or stalk domain, followed by immunoprecipitation with anti-eIF4G antibody (Fig. 6*E*), both WT CCHFV N protein and its head domain copurified with eIF4G. Taken together, it is clear that CCHFV N protein binds to eIF4A through its head domain, which likely facilitate the mRNA translation with the assistance of viral mRNA 5′ UTR.

### CCHFV N protein binds to the hairpin structure of S-segment mRNA 5′UTR

**Figure 7:**
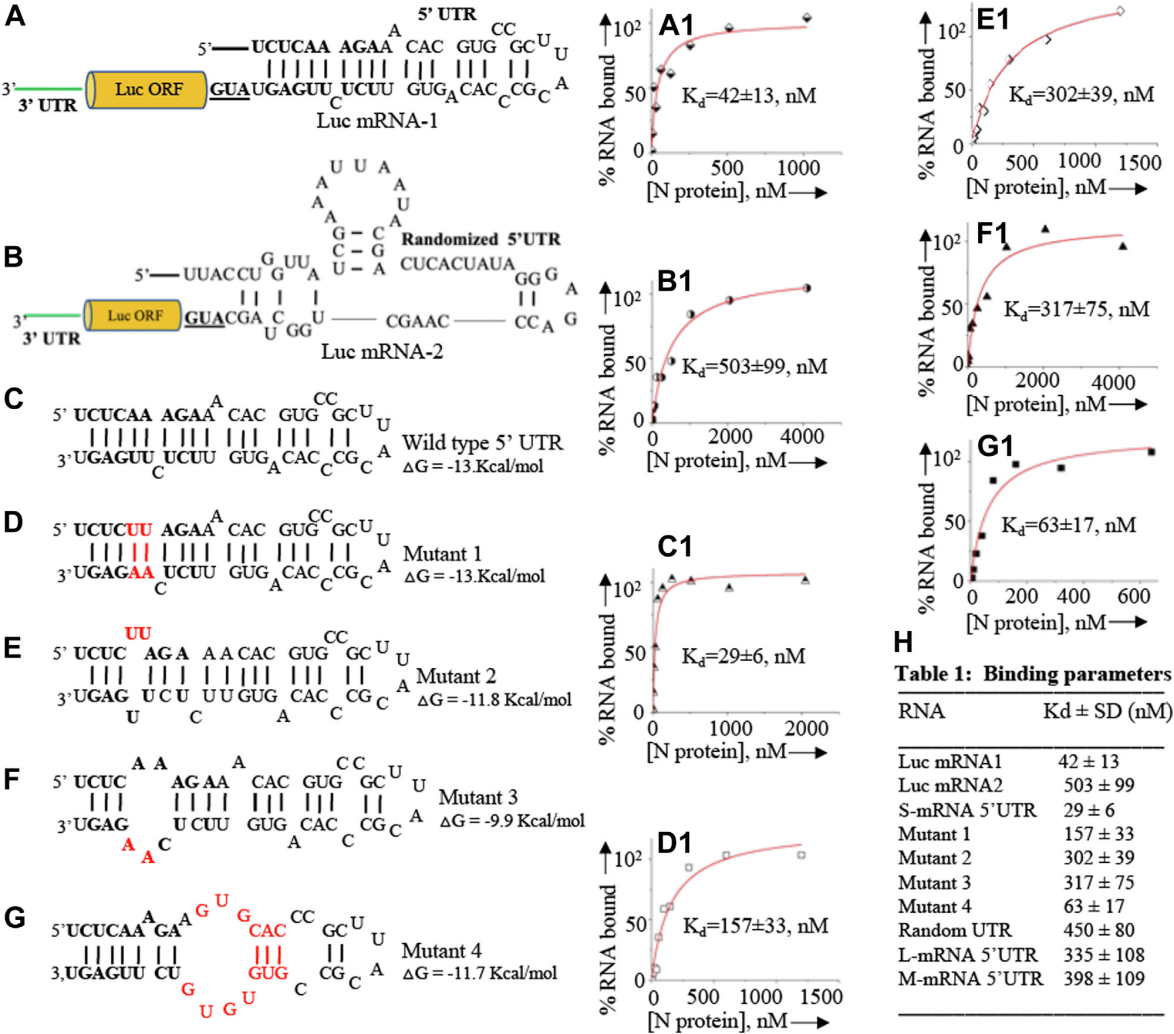
***In vitro*****filter binding analysis.***A and B*, pictorial presentation of Luc mRNA-1 (*A*) and Luc mRNA-2 (*B*) showing the fusion of S-segment mRNA 5′ UTR and randomized 5′ UTR, respectively, upstream of luciferase ORF. The truncated 3′UTR is shown in *green* line. The solid *black* line at the 5′ terminus represents the cap snatched sequence. *C*-*G*, secondary structures of WT S-segment 5′ UTR (*C*) and its mutants (*D*–*G*) are shown. The mutated nucleotides are shown in *red* color. The secondary structures were obtained by *mfold*, as mentioned in Experimental procedures. *A1*–*G1*, the interaction of purified CCHFV N protein with the synthetic RNA molecules shown in (*A*–*G*) was carried out using filter binding assay, as described in Experimental procedures. The binding profile of each RNA molecules in (*A*–*G*) is shown in corresponding (*A1*–*G1*). *H*, the dissociation constant (k_d_) for the binding of CCHFV N protein with all tested RNA molecules are shown.

We aimed to investigate whether the preferential translation of luciferase reporter mRNA, which mimics the structure of the S-segment mRNA (Fig. 2*A*), is linked to the binding of N protein to the 5′ UTR. We synthesized luciferase reporter mRNAs with either the WT S-segment mRNA 5′ UTR (Fig. 7*A*) or a randomized 5′ UTR (Fig. 7*B*) using a T7 transcription reaction, as described in the Experimental procedures. Both transcripts included a truncated 3′ UTR from the viral S-segment mRNA and a short sequence of 14 nucleotide upstream of the 5′ UTR, representing the cap-snatched sequence from host cell transcripts during cap-snatching mechanism of transcription initiation. Since the CCHFV N protein does not interact with the mRNA 5′ cap, the transcripts lacked a 5′ cap. The mRNAs were radioactively labeled during synthesis, as previously described (29, 31, 43). Using a filter binding assay, also detailed in the Experimental procedures, we analyzed the interaction of radiolabeled mRNAs with purified CCHFV N protein. As shown in Figure 7*A1*, the luciferase mRNA with the WT 5′ UTR exhibited high-affinity binding to the N protein (K_d_ = 42 ± 13 nM) compared to the luciferase mRNA with the randomized 5′UTR (K_d_ = 503 ± 99 nM). These results suggest that the preferential translation of luciferase mRNA (Fig. 2) is likely due to the high-affinity interaction between the N protein and the WT S-segment mRNA-derived 5′ UTR fused to the luciferase mRNA.

We then sought to pinpoint the specific region within the 5′ UTR sequence necessary for N protein binding. Our prior findings showed that the N protein binds selectively to the panhandle structure, formed by base pairing between the partially complementary nucleotides at the 5′ and 3′ termini of the S-segment vRNA (Fig. 1*C*) (29, 35). High-affinity binding specifically requires the base pairing of the terminal nine nucleotides of the 5′ end with the complementary 3′ end (29, 35). Notably, these terminally paired nucleotides of the vRNA panhandle are relatively conserved within the hairpin loop structure of the S-segment mRNA 5′ UTR (compare Fig. 1, *C* and *D*). We synthesized the S-segment mRNA 5′ UTR using T7 transcription and incorporated ^32^P-CTP in the RNA during synthesis. To investigate binding requirements, we introduced mutations into the stem of the hairpin structure. These mutations either preserved the base-paired stem structure (Mutant 1, Fig. 7*D*) or disrupted the stem's base pairing (mutants 2 and 3, Fig. 7, *E* and *F*). Filter-binding assays demonstrated that both the nucleotide sequence and the formation of the base-paired stem structure are essential for high-affinity N protein binding (compare Fig. 7*C1* with Figure 7, *D*1, *E1*, and *F1*). In contrast, mutations downstream of the stem region (Fig. 7*G*) only slightly affected binding affinity (compare Fig. 7*C1* with Fig. 7*G1*).

Additionally, filter binding analysis with a randomized S-segment mRNA 5′ UTR, as well as the 5′ UTRs derived from the L-segment and M-segment mRNAs (shown in Fig. 1), indicated poor binding to the N protein (Fig. 7*H*). Collectively, these findings suggest that similar to the vRNA panhandle, the stem structure formed by base pairing between the 5′ terminal nine nucleotides and their partially complementary counterparts in the S-segment mRNA 5′UTR is crucial for N protein binding.

### Randomization of 5′ UTR disrupts the translation of viral mRNA in cells

**Figure 8:**
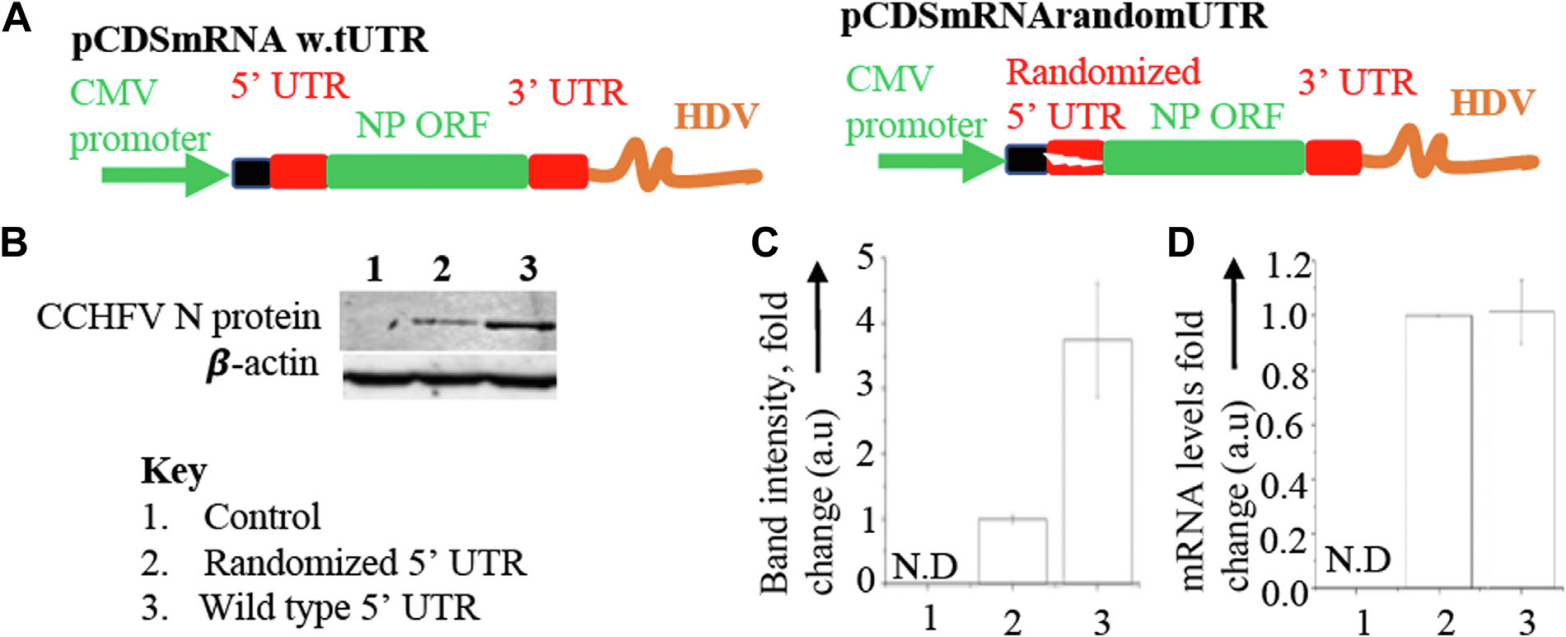
**Role of 5’ UTR in the translation of viral mRNA.***A*, schematic diagram of the plasmids pCDSmRNAw.tUTR and pCDmRNArandomUTR. *B*, HeK293T cells were mock transfected (lane 1) or transfected with either pCDSmRNArandomUTR plasmid (lane 2) or pCDSmRNA w.tUTR plasmid (lane 3), followed by examination cell lysates by Western blot analysis using anti-His tag antibody or anti β-actin antibody. *C*, fold changes in the band intensities of the CCHFV N protein are shown. *D*, real time PCR analysis showing relative S-segment mRNA levels in transfected cells.

The viral mRNAs encoded by the S, L, and M segments of CCHFV genome harbor a highly conserved 5′ UTR of ∼50 nucleotides in length. The 3′ UTR is truncated and lacks the 3′ poly(A) tail. We next wanted to determine whether the 5′UTR of S-segment mRNA regulates the intrinsic steady state levels of N protein in cells. We generated a pCDSmRNA w.tUTR construct that expressed the CCHFV S-segment mRNA harboring the WT 5′ UTR sequence and 14 nucleotide long RNA sequence upstream of the 5′UTR, representing the cap-snatched sequence. The 3′ end is truncated by selective incorporation of the HDV sequence (Fig. 8*A*). The exact sequence of the truncated 3′ UTR was previously identified by 3′RACE experiment (31). Another plasmid pCDSmRNArandomUTR expresses the similar mRNA except the 5′ UTR sequence was randomized. HeK293T cells were transfected with these plasmids and both N protein expression (Fig. 8*B*) and mRNA levels (Fig. 8*D*) were examined by Western blot and real time PCR analysis, respectively. It is evident from Figure 8, *A*–*D* that randomization of 5′ UTR abrogated the translational efficiency of the mRNA, leading to significantly low N protein levels in transfected cells. The mRNA levels were not affected by the randomization of the 5′UTR. This suggests that 5′ UTR regulates the intrinsic steady state levels of N protein in cells.

### CCHFV N protein facilitates the ribosome engagement on viral mRNA with the assistance of 5′ UTR

The CCHFV N protein binds to the viral mRNA 5′ UTR *via* its stalk domain and interacts with eIF4A through its head domain. The eIF4A forms a strong complex with eIF4G, which then recruits the 40S ribosomal subunit by directly interacting with eIF3 (Fig. 1*A*). This indicates that the simultaneous binding of the CCHFV N protein to the viral mRNA 5′ UTR and eIF4A through its stalk and head domains, respectively, may create a ribosome landing pad, facilitating the selective engagement of ribosomes on viral transcripts. To test this hypothesis indirectly, we asked whether WT S-segment mRNA is preferentially engaged with host ribosomes compared to S-segment mRNA with a randomized 5′ UTR. HEK293T cells were transfected with either the pCDSmRNAw.tUTR plasmid or the pCDSmRNArandomUTR plasmid, expressing S-segment mRNA with WT and randomized 5′ UTRs, respectively, as shown in Figure 8*A*.

**Figure 9:**
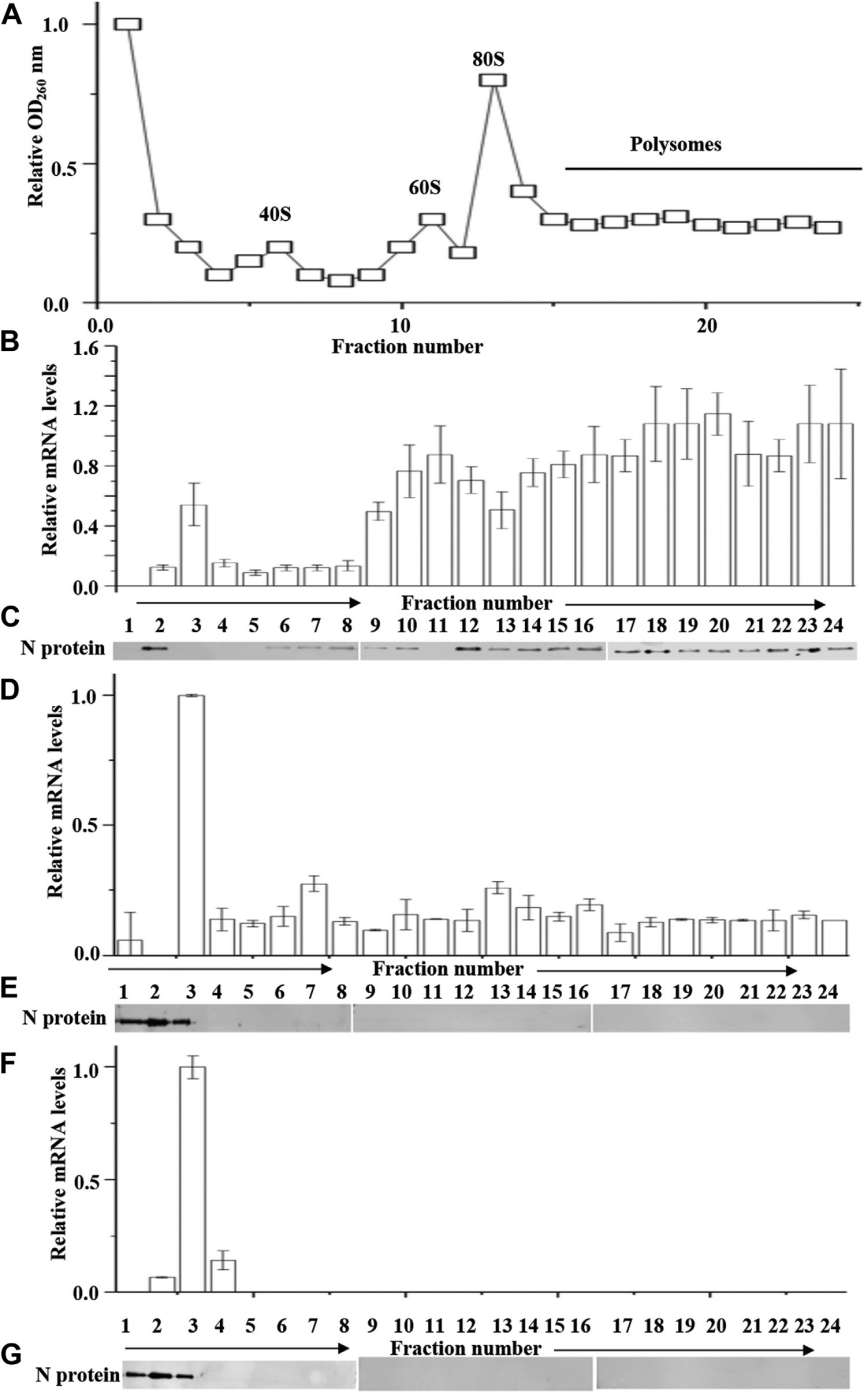
**Sucrose density gradient analysis.***A*, sucrose density gradient centrifugation and ribosome sedimentation profiles of HEK293T cell lysates expressing WT CCHFV S-segment mRNA from the transfected plasmid, as discussed in the text. *B*, real time PCR analysis showing the relative levels of WT S-segment mRNA in each fraction of the density gradient. *C*, Western blot analysis showing the presence of CCHFV N protein in the gradient fractions. *D*, HEK293T cells lysates expressing the S-segment mRNA with randomized 5′ UTR were fractionated on the 5 to 40% sucrose gradient as mentioned in the text. Real time PCR analysis showing the relative levels of S-segment mRNA harboring randomized 5′ UTR. *E*, gradient fractions were examined by Western blot analysis showing the presence of CCHFV N protein in each gradient fraction. *F*, sucrose density gradient centrifugation of synthetic WT CCHFV S-segment mRNA. Real time PCR showing the relative levels of S-segment mRNA in gradient fractions. *G*, the bacterially expressed and purified C-terminally His tagged CCHFV N protein was fractions on sucrose density gradient. Gradient fractions were examined by Western blot analysis for the presence of CCHFV N protein in each fraction.

Cell lysates were fractionated on a 5 to 40% sucrose gradient, and ribosome sedimentation profiles were recorded based on absorbance at 260 nm (A260) (Fig. 9*A*). Total RNA was purified from gradient fractions, and viral S-segment mRNA was quantified by real-time PCR. The relative abundance of S-segment mRNA was plotted against gradient fractions. Additionally, each fraction was subjected to Western blot analysis to identify the CCHFV N protein. Figure 9*B* shows that WT S-segment mRNA was predominantly present in the polysome fractions of the sucrose gradient, and N protein was observed in most of the gradient fractions (Fig. 9*C*). In contrast, S-segment mRNA with a randomized 5′ UTR was mostly found in fraction 3 towards the top of the gradient (Fig. 9*D*). Interestingly, the N protein expressed from this transcript was also present at the top of the gradient (Fig. 9*E*).

To gain insight into the sedimentation of purified N protein and synthetic S-segment mRNA in the sucrose gradient, we expressed and purified the CCHFV N protein from bacteria, as previously reported (44, 45). The S-segment mRNA was synthesized by a T7 transcription reaction, as previously reported (44, 45). The purified N protein and synthetic S-segment mRNA were also fractionated in the same sucrose gradient. Figure 9, *F* and *G* show that both N protein and S-segment mRNA were mostly observed in the top fractions of the gradient.

In summary, Figure 9 demonstrates that ribosomes were efficiently engaged with WT S-segment mRNA, as evidenced by its presence in polysome fractions of the gradient (Fig. 9*B*). This efficient ribosome engagement is consistent with the efficient translation of WT mRNA, generating relatively higher N protein levels in cells (Fig. 8). Since N protein binds to the WT 5′ UTR through its stalk domain with high affinity, it is likely that it remains associated with mRNA engaged in polysomes and continuously facilitates ribosome loading through the 5′ UTR. Unlike the N protein expressed from mRNA with a randomized 5′ UTR, the sedimentation of N protein from WT S-segment mRNA to polysome fractions suggests that its comigration to these fractions is not due to coupling with ribosome–mRNA complexes.

Randomization of the 5′ UTR impairs the efficient N protein–mediated ribosome engagement on S-segment mRNA, as evidenced by its presence in the top fractions of the gradient where synthetic mRNA devoid of ribosome engagement sedimented (compare Fig. 9, *D* and *F*). This poor ribosome engagement likely results in the lower levels of N protein in cells from the transcript with the randomized 5′ UTR (Fig. 8). Previously, we reported that CCHFV N protein poorly binds to the randomized 5′ UTR, which is supported by the observation that it was present in the top fractions of the gradient where purified N protein sedimented (compare Fig. 9, *E* and *G*). The absence of CCHFV N protein in the polysome fractions (Fig. 9*E*) and its poor binding to the randomized 5′ UTR (29, 35) suggest that S-segment mRNA containing the randomized 5′ UTR is not translated *via* the CCHFV N protein–mediated translation strategy.

### Discussion

The rapid increase in viral load within infected hosts is driven at the molecular level by the swift replication of viral genomes and the efficient production of viral proteins in the host cell. Viruses have developed selfish strategies to favor the translation of their own mRNAs in the cytoplasm of host cells, where cellular transcripts compete for the same translation machinery. For instance, picornaviruses suspend cap-dependent translation initiation, and herpes simplex virus degrades cellular mRNAs to eliminate competition from host cell transcripts (35). Similarly, the nsp1 protein of SARS coronavirus suppresses host gene expression by promoting the degradation of host cell mRNA and inactivating the translational activity of the 40S ribosomal subunit (18). However, it is intriguing how negative-strand RNA viruses like CCHFV or hantaviruses, which do not induce translation shutoff or cytopathic effects in host cells (6), manage to efficiently synthesize viral proteins during infection. Our findings suggest that these viruses likely use their nucleocapsid proteins to preferentially engage the host translation machinery at the viral mRNA 5′ UTR for efficient viral protein synthesis.

The engagement of the 40S ribosomal subunit at the mRNA 5′ cap is the rate-limiting step in eukaryotic translation. In eukaryotic cells, mRNA translation begins when the eIF4F complex, composed of the initiation factors eIF4A, eIF4E, and eIF4G, assembles on the mRNA cap. This eIF4F complex, also known as the ribosome loading pad, is crucial for translation initiation as it forms the first contact with the 40S ribosomal subunit *via* the interaction between eIF3 and eIF4G (Fig. 1*A*).

The circularization of eukaryotic mRNA during polysome formation stabilizes the mRNA and is necessary for efficient translation to rapidly increase intrinsic protein levels in the cytoplasm. This circularization is achieved through the interaction between the eIF4F complex and the poly(A)-binding protein (Fig. 1*A*). Interestingly, CCHFV mRNAs are truncated at the 3′ terminus and lack the 3′ poly(A) tail, raising questions about how viral proteins are efficiently synthesized in infected cells for the rapid buildup of the viral load without mRNA circularization.

The translation of luciferase reporter mRNA, which mimics the structure of the CCHFV S-segment mRNA, was significantly enhanced in cells co-expressing the CCHFV N protein (Fig. 2). However, when the 5′ UTR sequence was randomized, the N protein co-expression did not enhance the translation of the reporter mRNA in cells, confirming that the N protein requires the 5′ UTR to support translation. The CCHFV N protein consists of head and stalk domains, with the stalk domain specifically binding to the stem of the hairpin-like secondary structure of the 5′ UTR (Fig. 7), a binding necessary for facilitating the translation of the downstream ORF (31).

To determine if the N protein requires any components of the eIF4F complex to facilitate mRNA translation with the viral 5′ UTR, chemical inhibitors rapamycin and silvestrol were used to inhibit eIF4E and eIF4A, respectively. Figure 2, *B*–*D* show that chemical inhibition of eIF4E did not affect the translation of luciferase reporter mRNA mimicking the S-segment mRNA when N protein was co-expressed in cells, indicating that N protein does not require eIF4E for mRNA translation. However, chemical inhibition of eIF4A (Fig. 3) inhibited the translation of luciferase reporter mRNA in a dose-dependent manner, regardless of N protein co-expression (Fig. 3, *B*–*D*). This demonstrates that N protein requires eIF4A to facilitate mRNA translation with the assistance of the S-segment mRNA 5′ UTR.

**Figure 10:**
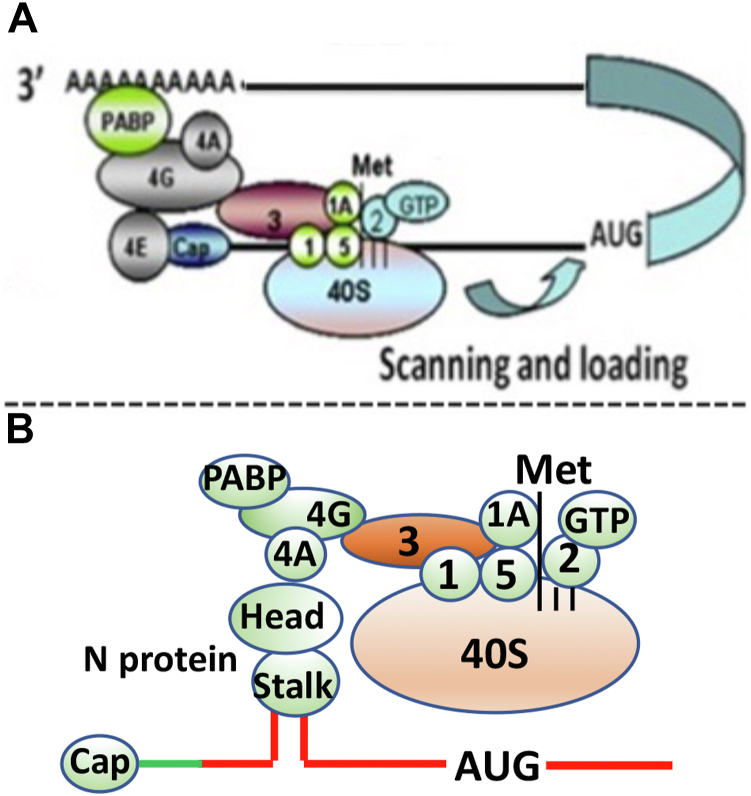
**A model for CCHFV N protein-mediated translation initiation.***A*, translation initiation of eukaryotic mRNA. *B*, a model for CCHFV N protein–mediated translation initiation.

Similarly, N protein failed to rescue luciferase reporter mRNA from translational shutdown caused by the proteolytic cleavage of eIF4G (Fig. 4, *A* and *B*), clearly indicating the necessity of eIF4G for N protein–mediated translation. This observation aligns with previously reported similar findings (31). Immunoprecipitation studies revealed that N protein directly binds to eIF4A *via* the head domain (Figs. 5 and 6). Since eIF4A forms a strong complex with eIF4G, which in turn interacts with eIF3, a component of the 43S pre-initiation ribosome complex, it is highly likely that the simultaneous binding of N protein to both the S-segment mRNA 5′ UTR and eIF4A through the stalk and head domains, respectively, facilitates ribosome loading with the assistance of eIF4G (Fig. 10). This also explains why eIF4G is required for N protein–mediated translation without a direct interaction between them.

To investigate if the N protein–mediated translation mechanism enhances the translation of viral mRNA in the host cell, the translation efficiency of WT S-segment mRNA harboring the WT 5′UTR was compared to S-segment mRNA with a randomized 5′UTR (Fig. 8). The WT S-segment mRNA encoding the N protein produced significantly higher translation products than the mRNA with a randomized 5′UTR (Fig. 8). Sucrose density gradient centrifugation analysis showed that WT S-segment mRNA was heavily engaged with ribosomes, comigrating with polysomes to the high-density zone in the gradient (Fig. 9). The N protein also comigrated with its mRNA towards the high-density zone (Fig. 9), suggesting that the N protein likely remains associated with the hairpin structure of WT 5′UTR through its stalk domain and continuously facilitates ribosome loading, promoting polysome formation and enhancing protein production. In contrast, most S-segment mRNA with a randomized 5′UTR was largely free from ribosome engagement (Fig. 9), explaining the lower protein production from this transcript (Fig. 8). Since the N protein does not bind to the 5′ UTRs of the L- and M-segment mRNAs, it is unlikely that the N protein–mediated translation strategy affects the translation of the viral RNA-dependent RNA polymerase or the glycoproteins Gn and Gc.

In summary, our findings suggest that CCHFV has likely evolved a unique strategy to enhance the translation of viral S-segment mRNA using its highly conserved 5′ UTR and N protein. The N protein sequesters a population of eIF4A–eIF4G complexes that remain dedicated to translating viral S-segment mRNA with the aid of its 5′ UTR, thus avoiding competition with host cell transcripts for the eIF4F complex to initiate translation. This unique translation mechanism is illustrated in the model (Fig. 10).

The interaction of the N protein with both the 5′ UTR and eIF4A is essential for the N protein–mediated translation mechanism. This makes the N protein–5′ UTR and N protein–eIF4A interactions attractive targets for the development of antiviral therapeutics. To our knowledge, there are currently no reportsspecifically targeting the interaction between eIF4A and a viral protein for antiviral drug design. However, eIF4A inhibitors have been shown to exhibit antiviral properties. For example, silvestrol and rocaglamides, two compounds derived from the Aglaia plant family, have demonstrated potent antiviral effects through inhibition of eIF4A (46). Silvestrol inhibits viral protein synthesis by clamping the 5′-UTRs of mRNAs onto the surface of the RNA helicase eIF4A (47). This mechanism has shown efficacy against various RNA viruses, including hepatitis E virus (46, 48, 49). Studies indicate that silvestrol not only reduces viral replication but also minimizes toxicity compared to other inhibitors, making it a promising therapeutic candidate. Rocaglamides on the other hand have shown broad-spectrum antiviral activity, notably against coronaviruses, without promoting viral resistance due to their targeting of a host factor (eIF4A) rather than viral proteins (47). Therefore, eIF4A inhibitors may potentially disrupt the N protein–eIF4A interaction, offering a promising avenue for antiviral development against viruses like CCHFV.

## Experimental procedures

### Cells and transfection

HEK293T cells (BEI Resources) were maintained in Dulbecco's modified Eagle's medium containing 10% fetal bovine serum and penicillin-streptomycin (100 μg/ml) in a CO_2_ incubator. All plasmid DNA transfections were performed using TurboFect transfection reagent (Thermo Fisher Scientific) according to the manufacturer's instructions. RNase A was from Invitrogen (Cat# 12091021). Anti-eIF4G antibodies were from Cell Signal Technologies (Cat# 2498S and 2469S).

### Plasmid construction

Straightforward cloning techniques were used to generate the constructs used in this study. Briefly, PCR was used to fuse the DNA sequence encoding the truncated CCHFV S-segment mRNA 3′ UTR (147 nucleotides) at the 3′ terminus of luciferase ORF. The DNA sequences encoding the CCHFV S-segment mRNA 5′ UTR (strain 10,200) and HDV were then fused upstream of AUG codon and downstream of the 3′ truncated UTR of the resulting PCR product, respectively. The resulting final PCR product was cloned 15 nucleotides downstream of transcription initiation site in pCDNA 3.1 (+) backbone between SacI and XhoI restriction sites to generate the pCDLuc3 construct, used in Figure 2, Figure 3, Figure 4. Same strategy was used for the construction of pCDLuc4 construct, except the 5′ WT UTR was replaced by the randomized UTR sequence. The plasmids pETCCHFV NP, pEThead, pETstalk, pETSNVNP, used for the expression and purification of WT CCCHFV N protein, head domain, stalk domain, and SNV N protein, respectively, in bacteria were generated as previously reported (29). The plasmids pTriEx CCHFV NP, pTriExhead, pTriExstalk, pTriEx SNV NP, used for the expression of CCHFV N protein, head domain, stalk domain, and SNV N protein in mammalian system were generated as previously reported (29). The plasmid pCDSmRNAw.tUTR was generated by fusing the HDV sequence at the 3′ terminus of DNA segment encoding the CCHFV S-segment mRNA with a truncated 3′ UTR, using PCR. The resulting final PCR product was then cloned 15 nucleotides downstream of transcription initiation site in pCDNA 3.1 (+) backbone between SacI and XhoI restriction sites to generate the pCDSmRNAw.tUTR. The same approach was used to construct the pCDSmRNArandomUTR plasmid, with the difference that the WT 5′ UTR sequence was replaced by a randomized 5′ UTR sequence. The plasmid pF/HRV-16 2A was generated as previously reported (17).

### Expression and purification of CCHFV-N protein, stalk domain, head domain, and SNV N protein in bacteria

The plasmids pETCCHFV NP, pEThead, pETstalk, pETSNVNP expressing C-terminally His tagged CCHFV-NP, head domain, stalk domain, and SNV N protein, respectively, were transformed into *Escherichia coli* Rosetta (DE3) cells (Novagen). The bacterial cultures were induced by 0.5 mM IPTG when OD at 600 nm reached 0.6. The cultures were grown for additional 20 h at 16 °C. Cell lysates were processed for the purification of recombinant proteins using NiNTA chromatography, following a native purification protocol, as previously reported (44, 45).

### Luciferase assays

HEK293T cells at a density of ∼30,000 cells per well were platted 1 day before the experiment. Celle were transfected with protein expression plasmids of interest using Turbofact transfection reagent (Promega), following manufacturer's protocol. Thirty-six hours post-transfection, cells were again transfected with either pCDLuc3 or pCDLuc4 construct, followed by treatment with increasing concentrations of either rapamycin or silvestro 5 hours post transfection. Cells incubate in CO2 incubator for 12 h post chemical treatment, followed by equilibration at room temperature for 30 min. The luciferase reagent (Cat# E2920, Promega) was thawed and equilibrated at room temperature for 30 min before use. The luciferase reagent was added to each test well of the 96-well plate, following the manufacturer's instructions. The plate was then incubated for 20 min at room temperature with continuous shaking on a rocker at low speed to allow the cell lysis. The luciferase signal was measured using the GloMax Navigator (Promega). It must be noted that this approach in which cell lysates are not removed from the wells for the measurement of luciferase signal has significantly improved the assay sensitivity and the quality of results. The observed fold changes in treated samples related to the respective controls were significantly improved by this approach in comparison to previous methods where the cell lysates were pelleted down and the supernatant was examined for luciferase activity (31).

### Western blot and antibodies

Cells were washed once with PBS and lysed with radioimmunoprecipitation assay buffer supplemented with protease and phosphatase inhibitor cocktails (Roche), as previously reported (50). Clarified cell extracts were mixed with equal volume of 2×SDS loading buffer and boiled at 95 °C for 5 min. Proteins were separated by SDS-PAGE and transferred to polyvinylidene di fluoride membrane (Millipore). The membrane was blocked with 5% nonfat milk in PBST buffer (1× PBS, 0.05% Tween 20) followed by incubating with primary and secondary antibodies diluted in blocking buffer. The primary antibodies for eIF4A (Cat# 2013S), eIF4G (Cat# 2469S), eIF4E (Cat# 2067S), phosphor-4EBP1 (Cat# 2855), 4EBP1 (Cat# 9452), and ant-His tag antibody (Cat# 2366) were from Cell Signaling Technologies. The antibody for CCHFV N protein (ab190657) was from abcam. The secondary antibodies, donkey anti-rabbit (cat# 926803), goat anti-rat (cat# 9256807), donkey anti-mouse (Cat# 9266807) were from LI-COR. The blots were scanned using the Odyssey CLx imaging system from LI-COR. It must be noted that the antibodies for eIF4E, eIF4A, and eIF4G showed background. In addition, the CCHFV N proteins, head domain, stalk domain, and SNV N protein showed mild degradation in cells. The background and degradation products were cropped from the images for better presentation.

### Coimmunoprecipitation

Coimmunoprecipitation was carried out as previously described (51). Briefly, HEK293T cells were cotransfected with required plasmids. Cells were lysed with NP-40 lysis buffer (50 mM Tris–HCl pH 7.5, 150 mM NaCl, 0.5% NP-40, 10% glycerol, 1 mM EDTA), supplemented with protease and phosphatase inhibitor cocktails (Roche). The lysates were incubated with 4E1Rcat and RNase A at room temperature for 1 hour, as mentioned in Figures 5 and 6. Ten percent of the clarified cell lysates were saved as input. The remaining cell lysates were incubated with 1 μg of required antibody for 4 h at 4 °C with gentle rotation. The antigen–antibody complexes were captured with 40 μl of protein G agarose beads (50% slurry) by continuous rotation for 1 hour at 4 °C. The beads were washed four times with lysis buffer, resuspended in 1×SDS loading buffer, and boiled at 95 °C for 5 min. After brief centrifugation, the supernatants were loaded into SDS PAGE gel. Coimmunoprecipitation using purified protein was carried out similarly. Briefly, the purified proteins of interest were mixed at a concentration of 143 μM each in 1× PBS, followed by incubation at room temperature for 1 hour to allow the protein–protein interaction. The resulting mixture was incubated with 1 μg of antibody of interest, followed by the capture of antigen–antibody complexes as mentioned above. The immunoprecipitated material was separated on SDS PAGE gel and examined by Western blot analysis, using appropriate antibodies.

### m7G sepharose pulldown

Cells were lysed using lysis buffer (10 mM Tris–HCl, pH 7.6, 140 mM KCl, 4 mM MgCl_2_, 1 mM DTT, 1 mM EDTA, and protease inhibitors) supplemented with 1% NP-40. The resulting cell lysates (750 μg protein in 500 μl) were incubated overnight at 4 °C with 50 μl of mRNA cap analog m^7^GTP-sepharose (Jena Bioscience) in lysis buffer, maintained under gentle and continuous agitation. After incubation, the Sepharose beads were washed three times with lysis buffer containing 0.5% NP-40. Finally, the beads were boiled in 100 μl of 1× SDS-PAGE loading dye, briefly centrifuged, and the resulting supernatants were analyzed by Western blotting using appropriate antibodies.

### Sucrose density gradient fractionation

Sucrose density gradient fractionation of Ribosomal subunits and polysomes in HEK293T cell lysates was carried out as previously reported (52). Briefly, HEK293T cells seeded in 10 cm dish were transfected with plasmids pCDSmRNAw.tUTR. and pCDSmRNArandomUTR, expressing S-segment mRNA harboring WT and randomized 5′ UTR, respectively. Cells were treated with cycloheximide (100 μg/ml final concentration) for 5 min at 24 h post-transfection. Cells were washed twice with ice-cold PBS containing 100 μg/ml cycloheximide. Cells were scraped in 5 ml of ice-cold PBS containing 100 μg/ml cycloheximide and centrifuged at 500*g* for 5 min. Cell pellet was resuspended in 500 μl of hypotonic buffer (5 mM Tris–HCl pH 7.5, 2.5 mM MgCl_2_, 1.5 mM KCl) supplemented with 1× protease inhibitor cocktail (Roche), 100 μg/ml cycloheximide, 2 mM DTT, 100 units of RNAase inhibitor, followed by the addition of Triton X-100 and sodium deoxycholate to a final concentration of 0.5% to solubilize ribosomes for 20 min at 4 °C. Cell lysates were centrifuged at 160,00*g* for 15 min at 4 °C and supernatants were layered on linear sucrose gradient (5%–40%) centrifuged at 37,000 rpm for 3.5 h at 4 °C. The sucrose gradient fractions were collected and optical density at 260 nm was measured.

### T7 transcription for RNA synthesis

The DNA segment encoding the Luc mRNA-1 and Luc mRNA-2 (Fig. 7, *A* and *B*) were PCR amplified from pCDLuc3 and pCDLuc4 plasmids, respectively, using two opposing primers. The forward primer contained a flanking T7 promoter. For the synthesis of shorter RNA molecules, two complementary DNA oligos having appropriately positioned T7 promoter were annealed together and subjected to twenty rounds of PCR amplification to fill the incomplete ends, if any. The resulting PCR products encoding WT S-segment mRNA 5′ UTR, mutants 1 to 4 (Fig. 7, *C*–*G*), randomized 5′UTR (Fig. 1*E*), L and M-segment derived 5′ UTRs (Fig. 1, *F* and *G*) were gel purified and used in T7 transcription reaction for the synthesis of RNA of interest, as previously reported (16, 53, 54, 55, 56). The RNA molecules were radiolabeled during synthesis using [α^32^P] CTP as previously reported (16, 53, 54, 55, 56). The DNA templates were digested using DNAse1. The radiolabeled RNA was purified using trizol reagent and RNeasy purification kits (Qiagen), as previously reported (16, 53, 54, 55, 56).

### RNA filter-binding analysis

Interaction of bacterially expressed and purified CCHFV N protein with the RNA of interest (Fig. 7) was studied by filter-binding assay, as previously reported (29, 35). Briefly, the desired RNA molecules were synthesized *in vitro* using T7 RNA polymerase and radiolabeled with [α^32^P] CTP during synthesis, as mentioned above. All binding reactions were carried out in RNA-binding buffer (40 mM Tris–HCl [pH 7.4], 80 mM NaCl, 20 mM KCl, 1 mM DTT) at a constant concentration of RNA with increasing concentrations of N protein. Reaction mixtures were incubated at room temperature for 30 to 45 min and filtered through nitrocellulose membranes under vacuum. Filters were washed with 5 ml of RNA-binding buffer and dried. The amount of RNA retained on the filter at each input concentration of N protein was measured by quantifying the radioactive signal, using a scintillation counter. A binding profile was generated by plotting the radioactive signal along Y-axis and N protein concentration along X-axis. The percentage of bound RNA at each input concentration of N protein was calculated using the Equation 1.

(1) $$\text{Percentage}\;\text{of}\;\text{bound}\;\text{RNA}=\Delta R\;/\Delta R_{\max}*100$$

Where ΔR is the change in radioactive signal at each addition of N protein. ΔR_max_ is the same parameter when the RNA is totally bound to the N protein. *ΔR*_*max*_ was calculated by simply averaging the radioactive signal of saturating data points. The percentage of bound RNA obtained from Equation 1 was plotted verses input N protein concentration, and the resulting data points were fit to a dose-response equation using the program Origin 6 (Microcal). The apparent dissociation constant (*Kd*) corresponded to the concentration of N protein required to obtain the half-saturation in the fitted binding curve, assuming that the complex formation obeys a simple bimolecular equilibrium.

### RNA secondary structure analysis

We used mFold to analyze the most probable secondary structures of RNA molecules shown in Figures 1 and 7, as previously reported (57). The parameters were adjusted to a salt concentration of 1M NaCl and the folding temperature of 37 °C. While examining the suboptimal secondary structures predicted by mFold, we determined the P-num values for each of the nucleotides in the RNA, as previously reported (29, 35). The P-num value of a particular nucleotide in the RNA sequence represents the number of potentially stable pairing partners for that nucleotide elsewhere in the same RNA molecule. Viewing the P-num values from the context of suboptimal folds, the structures composed of low P-num nucleotides still form and are composed of bases with the fewest alternative pairing partners (58).

### Real time PCR

Total cellular RNA was purified using RNeasy Kit (Qiagen) and reverse transcribed using M-MLV Reverse Transcriptase (invitrogen) according to manufacturer's instructions. Real time PCR reactions were performed on ABI 7500 real time PCR system (Applied Biosystems), using SYBR green PCR master mix (Roche). Each reaction was performed in triplicates. The mRNA levels of a housekeeping gene β-actin were quantified as an internal control. The relative quantification method was used for data analysis as previously reported (51). The primers used for the quantification of β-actin mRNA, luciferase mRNA, and CCHFV S-segment RNA have been reported in our previous publication (51). Real time PCR analysis of CCHFV S-segment mRNA purified from sucrose gradient fractions (Fig. 8) was carried out similarly. Briefly, HEK293T cells lysates containing CCHFV S-segment mRNA harboring either WT or randomized 5′ UTR were fractionated on 5 to 40% sucrose gradient. The total RNA was purified from each gradient fraction using RNeasy kit (Qiagen). The RNA from each column was eluted in equal volume of elution buffer, and equal volume of eluted RNA from each fraction was subjected to real time PCR analysis using a primer set targeted to CCHFV S-segment mRNA.

## Data availability

All the data generated or analyzed during the study are included in the manuscript.

## Conflict of interest

The authors declare that they have no conflicts of interest with the contents of this article.
